# Epigenetic Silencing of STAT3-Targeted miR-193a, by Constitutive Activation of JAK/STAT Signaling, Leads to Tumor Progression Through Overexpression of YWHAZ in Gastric Cancer

**DOI:** 10.3389/fonc.2021.575667

**Published:** 2021-02-25

**Authors:** Kuo-Liang Wei, Jian-Liang Chou, Yin-Chen Chen, Jie-Ting Low, Guan-Ling Lin, Jing-Lan Liu, Te-Sheng Chang, Wei-Ming Chen, Yung-Yu Hsieh, Pearlly S. Yan, Yu-Ming Chuang, Jora M. J. Lin, Shu-Fen Wu, Ming-Ko Chiang, Chin Li, Cheng-Shyong Wu, Michael W. Y. Chan

**Affiliations:** ^1^ Division of Gastroenterology, Chang Gung Memorial Hospital, Chiayi, Taiwan; ^2^ Department of Biomedical Sciences, National Chung Cheng University, Chiayi, Taiwan; ^3^ Instrument Center, Department of Research and Development, National Defense Medical Center, Taipei, Taiwan; ^4^ Department of Anatomical Pathology, Chang Gung Memorial Hospital, Chiayi, Taiwan; ^5^ Division of Hematology, Department of Internal Medicine, Comprehensive Cancer Center, The Ohio State University, Columbus, OH, United States; ^6^ Epigenomics and Human Disease Research Center, National Chung Cheng University, Chiayi, Taiwan; ^7^ Center for Innovative Research on Aging Society (CIRAS), National Chung Cheng University, Chiayi, Taiwan

**Keywords:** STAT3, epigenetics, miR-193a, YWHAZ, gastric cancer

## Abstract

**Purpose:**

The purpose of this study was to identify genes that were epigenetically silenced by STAT3 in gastric cancer.

**Methods:**

MBDcap-Seq and expression microarray were performed to identify genes that were epigenetically silenced in AGS gastric cancer cell lines depleted of STAT3. Cell lines and animal experiments were performed to investigate proliferation and metastasis of miR-193a and YWHAZ in gastric cancer cell lines. Bisulfite pyrosequencing and tissue microarray were performed to investigate the promoter methylation of miR-193a and expression of STAT3, YWHAZ in patients with gastritis (n = 8) and gastric cancer (n = 71). Quantitative methylation-specific PCR was performed to examine miR-193a promoter methylation in cell-free DNA of serum samples in gastric cancer patients (n = 19).

**Results:**

As compared with parental cells, depletion of STAT3 resulted in demethylation of a putative STAT3 target, miR-193a, in AGS gastric cancer cells. Although bisulfite pyrosequencing and epigenetic treatment confirmed that miR-193a was epigenetically silenced in gastric cancer cell lines, ChIP-PCR found that it may be indirectly affected by STAT3. Ectopic expression of miR-193a in AGS cells inhibited proliferation and migration of gastric cancer cells. Further expression microarray and bioinformatics analysis identified YWHAZ as one of the target of miR-193a in AGS gastric cancer cells, such that depletion of YWHAZ reduced migration in AGS cells, while its overexpression increased invasion in MKN45 cells *in vitro* and *in vivo*. Clinically, bisulfite pyrosequencing revealed that promoter methylation of miR-193a was significantly higher in human gastric cancer tissues (n = 11) as compared to gastritis (n = 8, p < 0.05). Patients infected with H. pylori showed a significantly higher miR-193a methylation than those without H. pylori infection (p < 0.05). Tissue microarray also showed a positive trend between STAT3 and YWHAZ expression in gastric cancer patients (n = 60). Patients with serum miR-193a methylation was associated with shorter overall survival than those without methylation (p < 0.05).

**Conclusions:**

Constitutive activation of JAK/STAT signaling may confer epigenetic silencing of the STAT3 indirect target and tumor suppressor microRNA, miR-193a in gastric cancer. Transcriptional suppression of miR-193a may led to overexpression of YWHAZ resulting in tumor progression. Targeted inhibition of STAT3 may be a novel therapeutic strategy against gastric cancer.

## Introduction

Gastric cancer is the third leading cause of cancer deaths worldwide. Despite the advance in cancer therapy, the 5-year survival rate of gastric cancer is still less than 30% (1). Gastric cancer usually developed progressively from several histological stages including gastritis, and intestinal metaplasia (2, 3). These lesions are highly related to the infection of *Helicobacter pylori* (*H. pylori*), a Gram-negative bacteria that colonizes human stomach, and is also one of the risk factors for gastric cancer. Particularly, patients infected with *cytotoxin-associated gene A* (*CagA*)-positive strain of *H. pylori*, in comparison with *CagA*-negative strain, have increased risk of developing atrophic gastritis, as well as gastric cancer (4, 5). This may be attributed to the increased cytokine expression and activation of JAK/STAT signaling, resulting in robust inflammatory responses in the tumor microenvironment (6, 7).

JAK/STAT signaling is known to be involved in cancer development. Upon binding of IL-6 to its receptor, Janus kinase (JAK) is then activated for phosphorylation of STAT3, which is then dimerized and translocated into the nucleus, together with cofactors, to regulate transcription of its target genes. Previous studies including ours demonstrated that phosphorylated STAT3 can also suppress gene expression, by recruitment of DNMT1 (7–9). However, the role of STAT3 in epigenetic modifications of tumor suppressors is less explored.

MicroRNAs (miRNAs), small non-coding RNAs (approximately 22 nucleotide lengths), recognize 3’-untranslated regions (3’-UTR), open reading frame (ORF), or 5’-UTR of targeted mRNA to inhibit gene expression at post-transcriptional level. Numerous studies have demonstrated that miRNAs are aberrantly expressed in gastric cancer and play an important role in gastric cancer progression (10–12). The miR-193a that generates two mature miRNAs, miR-193a-3p, and miR-193a-5p was indicated as a tumor suppressor in various cancer, such as acute myeloid leukemia, thyroid carcinoma (13–15). Indeed, previous study showed that downregulation of miR-193a enhances Myeloid cell leukemia-1 (MCL1) expression and promotes gastric cancer proliferation, confirming the tumor suppressive role of miR-193a (16). However, the role of JAK/STAT3 signaling on epigenetic silencing of tumor suppressive miR-193a in gastric cancer has never been explored.

In this study, by high-throughput screening, we found that expression of miR-193a was epigenetic silenced by STAT3 activation. Epigenetic silencing of miR-193a led to overexpression of the adapter protein and metastatic regulator YWHAZ, resulting in gastric cancer progression.

## Materials and Methods

### Patient Samples

Ninety patient samples including gastritis (n = 9), tumor adjacent normal (n = 11), and cancer (n = 71) were obtained from Chang Gung Memorial Hospital, Chiayi, Taiwan (**Table 1**). For this cohort of gastric cancer patient samples, the median age at the time of diagnosis was 69 years (range, 47~87 years). Serum samples from 19 cancer patients were also obtained for methylation analysis of cell-free DNA (**Table 2**). All human subject assessments were approved by the Institutional Review Board (IRB) of the Chang Gung Memorial Hospital, Chiayi, Taiwan. The study was carried out in strict accordance with approved guidelines. Written informed consent was obtained from all participants.

**Table 1:** Summary of clinico-pathological data of patients’ samples.

	Gastritis (n = 8)	Adjacent normal (n = 11)	Cancer (n = 71)
Age			
Median	43	53	69
Range	26~77	23~78	46~87
Sex			
Male	5	5	50
Female	3	6	21
*H. pylori* infection			
Positive	3	2	43
Negative	5	9	28
Stage			
I			13
II			14
III			31
IV			13
Metastasis			
Yes			48
No			23

**Table 2:** Summary of clinico-pathological data of 19 gastric cancer patients with serum samples.

	Cancer (n = 19)
Age	
Median	67
Range	51~82
Sex	
Male	13
Female	6
*H. pylori* infection	
Positive	14
Negative	5
Stage	
I	5
II	3
III	9
IV	2
Metastasis	
Yes	12
No	7

### Cell Culture

Gastric cancer cell lines (AGS, KATO III, MKN28, MKN45, SNU1, and SNU16, purchased from ATCC, Manassas, VA, USA) were propagated in RPMI-1640 (Invitrogen, Carlsbad, CA, USA) containing 10% fetal bovine serum and incubated at 37°C under a humidified atmosphere containing 5% CO2. For DNA demethylation treatment, cells were treated with 0.5 μM 5’-aza-2’-deoxycytidine (5aza, Sigma, St. Louis, MO, USA) for 72 h, with or without 0.5 μM histone deacetylase (HDAC) inhibitor, trichostatin A (TSA, Sigma) for 12 h, or in combination. Cells were also treated with 2.5 μM of a STAT3 inhibitor RHD6 (9) for 72 h.

### Methyl-CpG Binding Domain-Based Capture and Sequencing (MBDCap-Seq)

Control and STAT3 depleted AGS cells were subjected to MBDCap-Seq to identify differentially methylated regions as previously described (17, 18). In brief, one microgram of sonicated DNA was incubated at room temperature on a rotator mixer in a solution containing 3.5 μg of MBD-Biotin Protein coupled to M-280 Streptavidin Dynabeads (Methyl Miner Kit, Invitrogen). Methylated DNA was enriched by collecting magnetic beads and washing three times with Bind/Wash Buffer. Library generation and 50-bp single-ended sequencing were performed on the Illumina HiSeq 2500 system according to the manufacturer’s standard protocol. All sequencing was performed at the sequencing core of the Ohio State University, Columbus, OH, USA. The sequencing data has been deposited in the Gene Expression Omnibus database (accession number: GSE154080).

### DNA Extraction, RNA Extraction, and Quantitative Reverse Transcription-PCR

The DNA was extracted using the Tissue & Cell Genomic DNA Purification Kit (Genemark, Taiwan). The DNA was eluted in 50 µl distilled water and stored at –20°C until use. Total RNA from cell lines was extracted using Trizol (Invitrogen) in accordance with the manufacturer’s protocol. Briefly, 1.0 μg of total RNA was treated with DNase I (Amplification Grade, Invitrogen), prior to reverse transcription. RNA was then reverse-transcribed, using oligo dT primers or a TaqMan microRNA reverse transcription kit (Applied Biosystems, Foster City, CA, USA) with primer specific to miR-193a (Applied Biosystems) or RNU48 (Applied Biosystems). Quantitative real-time RT-PCR was then performed using ABI Stepone real-time PCR system (Applied Biosystems). GAPDH and snoRNA (RNU48) were used as normalization controls for mRNA and miRNAs expression, respectively. All RT-PCR primer sequences are shown in **Table 3**. Relative expression levels were calculated using the comparative Ct method.

**Table 3:** Primer sequence used in this study.

RT-PCR	Sequence 5’-3’
*GAPDH* forward	CCCCTTCATTGACCTCAACTACAT
* GAPDH* reverse	CGCTCCTGGAAGATGGTGA
* Pri-miR193a* forward	GTCTTTGCGGGCGAGAT
* Pri-miR193a* reverse	TTGATGTCTGGGTCTTGGTTCT
* YWHAZ* forward	GTTTCCATGTCCCATGATCC
* YWHAZ* reverse	GGAAGCCACAATGTTCTTGG
Pyrosequencing	
* mir193a* forward	GGGTGTAGGATTAATTGGTTTATAAAGT
* mir193a* reverse	AACCCACCTCACCACTCCTTCTC
* mir193a* sequencing	ATTAATTGGTTTATAAAGTTTTAGT
UNIV biotin primer	AGCTGGACATCACCTCCCACAACG
Methylation Specific PCR	
* mir193a* MF	GGTGTATAGAGTCGGCGATC
* mir193a* MR	CGAAAACCGAAAAAAAAACG

### Bisulfite Conversion and Pyrosequencing

Bisulfite pyrosequencing was performed as described previously (19). Briefly, 0.5 µg of genomic DNA was bisulfite-modified using the EZ DNA Methylation Kit (Zymo Research, Orange, CA, USA), according to the manufacturer’s protocol. The bisulfite-modified DNA was subjected to PCR amplification using a tailed reverse primer in combination with a biotin-labeled universal primer. PCR and sequencing primers were designed using PyroMark Assay Design 2.0 (Qiagen GmbH, Hilden, Germany). The CpG site of miR193a was PCR amplified with specific primers (**Table 3**) in a 25 μl reaction using Invitrogen Platinum™ DNA Polymerases (Invitrogen). Prior to pyrosequencing, 1.5 μl of each PCR reaction was analyzed on 1% agarose gel. Pyrosequencing was performed on the PyroMark Q24 instrument (Qiagen) using Pyro Gold Reagents (Qiagen), according to the manufacturer’s protocol. The methylation level of 11 CpG sites, which are located +5 to +11 was measured. The methylation percentage of each cytosine was determined by dividing the fluorescence intensity of cytosines with the sum of the fluorescence intensity of cytosines and thymines at each CpG site. *In vitro* methylated DNA (Merck Millipore, Billerica, MA, USA) was included as a positive control for pyrosequencing.

### Quantitative Methylation-Specific PCR (qMSP)

Bisulfite-modified DNA was subjected to qMSP for miR-193a methylation analysis using ABI StepOne real time PCR system (Applied Biosystems) as previously described (20). Primers targeting the miR-193a promoter region were shown in **Table 3**. The amount of methylated miR-193a were determined by the threshold cycle number (Ct) for each sample against a standard curve generated by SssI treated DNA (Millipore, Billerica, MA, USA)-MSP cloned fragment of miR-193a.

### Chromatin Immunoprecipitation-PCR (ChIP-PCR)

Chromatin immunoprecipitation was performed according to Abcam X-ChIP protocol. In brief, AGS cells at 2 × 10^7^ were fixed with 1% formaldehyde for 10 min at room temperature. Cross-linking was stopped by the addition of glycine to a final concentration of 125 mM followed by a 5 min incubation. Cells were harvested and lysed with ChIP lysis buffer. DNA was then sheared by sonication (Diagenode Bioruptor) to 300–700 bp fragments. The cross-linked protein-DNA complex was immunoprecipitated using mouse anti-STAT3 (Cell Signaling, 124H6, #9139). DNA was purified using Qiaquick PCR purification kit (Qiagen). The amount of DNA was quantified by a SYBR green based real-time PCR (Applied Biosystems, StepOne), and the relative enrichment fold-change was calculated using delta Ct.

### Knockdown YWHAZ by shRNA

The shRNA of YWHAZ were acquired from the RNAi Core Facility (Academia Sinica, Taiwan). Briefly, 293T cells were transfected with shRNA (TRCN0000029093), pMDG, and pCMV-dR8.91 using ProFection Mammalian Transfection System (Promega) to prepare the shYWHAZ lentivirus. Infected gastric cancer cells were selected by incubating with 2 µg/ml puromycin (Sigma) for at least 2 days.

### Colony Formation Assay

Trypsinized cells (1,000 cells) were seeded and mixed in 1.5 ml of 0.35% top layer agar supplemented with DMEM with 10% FBS. This suspension was overlaid on the bottom layer of 0.5% agar in DMEM with 10% FBS in a six-well plate. Plates were allowed to solidify and then incubated at 37°C for around 3 weeks. Colony formation was monitored daily by microscopic observation. At the end of the experiments, the plates were stained with Iodonitrotetrazolium (INT) stain (Sigma) at 37°C for 48 h. The number of colonies were counted.

### Wound Healing Assay

For cell motility assay, the transfected cells were seeded in 6-cm plates and cultured to confluency. A single wound was created by scratching the cell by using a 20 µl sterile pipette tip. The cells were washed two times with PBS and incubated in culture medium. Images were taken at 0 and 12 h after wounding.

### Transwell Invasion Assay

Transwell chamber inserts with coated matrigel (Invitrogen) were used for cell invasion assays. The transfected cells (5 × 10^3^ cells) were seeded into the upper chamber of the 24 well with 500 µl serum free medium. Then 500 µl complete medium with 10% FBS was added to the bottom of the inserts, allowing cells to invade for 48 h. After incubation, the cells on the upper surface of the membrane were removed, whereas those on the lower filter surfaces were fixed and stained with Giemsa stain (Sigma). The number of migrated cells was counted under a microscope.

### Immunohistochemical Analysis on Gastric Cancer Tissue Microarray

Paraffin-embedded gastric cancer patients tissue microarray was prepared and retrieved from Chang Gung Memorial Hospital, Chiayi, Taiwan. The tissue microarray contained 60 samples of gastric cancer patients. The immunohistochemistry procedure followed a standard protocol, using an anti-human STAT3 and YWHAZ antibody (Cell signaling). All tissue microarray slides were examined and scored by pathologist.

### *In Vivo* Tumorigenicity Assay

A total of three, 6-week-old, NOD-SCID mice were obtained from the National Laboratory Animal Center, Taiwan. MKN45 cells (5 × 10^6^ for intraperitoneal injection and 1 × 10^6^ for subcutaneous injection) stably transfected with pcDNA3.1/YWHAZ or pcDNA3.1 were re-suspended in 0.1 ml of medium or medium/Matrigel (BD Bioscience, San Jose, CA, USA) mixture (1:1). For metastatic analysis, cells were intraperitoneally injected, and sacrificed after 5 weeks. Tumor nodules were observed in internal organs such as lung, liver, and stomach, for metastasis. For subcutaneous injection, cell suspension was injected subcutaneously into the flank of each mouse (day 0). Tumor size was measured daily with calipers in length (L) and width (W). Tumor volume was calculated using the formula (L × W^2^/2). At the end of experiment, all mice were sacrificed by cervical dislocation. All mice were kept under specific pathogen-free conditions using a laminar airflow rack, with free access to sterilized food and autoclaved water. All experiments were performed under license from Animal Experimentation Ethics Committee of the National Chung Cheng University.

### Statistical Analysis

Unpaired t-test was also used to compare parameters of the different groups. Overall survival was assessed using Kaplan-Meier analysis with the log-rank test. All statistical calculations were performed using GraphPad Prism 5 software (version 5.01). *P* values < 0.05 was considered to be statistically significant.

## Results

### STAT3-Mediated Epigenetic Silencing of miR-193a in Gastric Cancer Cell Line

**Figure 1:**
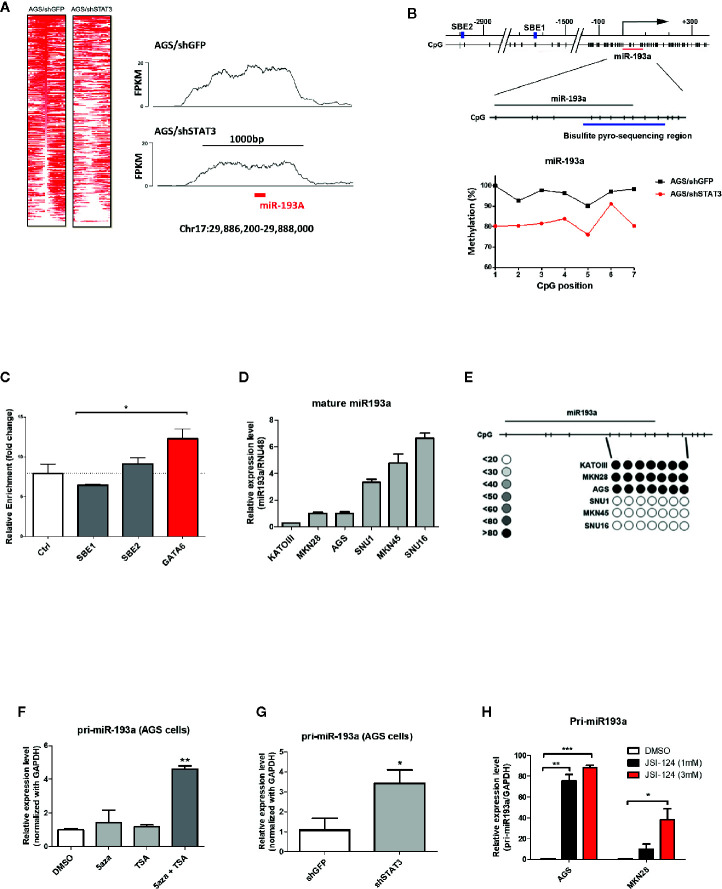
Identification of miR-193a as a STAT3-mediated hypermethylated target in gastric cancer cells. AGS gastric cancer cells infected with lentivirus expression control (AGS/shGFP) or shRNA against STAT3 (AGS/shSTAT3) were used to perform methylation and expression analysis. **(A)** Global methylation analysis of the parental AGS/shGFP or AGS/shSTAT3, by MBDcap-Seq. Left panel, heatmap showing differential methylated regions (DMR) in AGS/shSTA3 and AGS/shGFP cells. Right panel, representative histogram showing methylation level of chromosome region around miR-193a (chr17:29886200-29888000) in AGS control and STAT3 depleted cells. **(B)** Bisulfite sequencing of miR-193a methylation in AGS control and STAT3 depleted cells. Upper panel, schematic diagram showing the genomic map of miR-193a promoter, with the corresponding locations of CpG sites and the putative STAT3 binding site (SBE, blue box). The location of miR-193a (red line) and region for bisulfite pyrosequencing analysis (enlarged region) is also shown. Lower panel, scatter plot showing the methylation level of the seven CpG sites being interrogated in AGS control (AGS/shGFP) and STAT3 depleted cells (AGS/shSTAT3). **(C)** ChIP-PCR showing the binding of STAT3 to SBE1, SBE2 of the promoter region of miR-193a. Negative control region (Ctrl, -400 upstream of TSS) and positive control (GATA6) were also shown. Relative expression and methylation level of mature miR-193a in a panel of gastric cancer cell lines was also determined by **(D)** quantitative RT-PCR and **(E)** bisulfite pyrosequencing. Relative expression level of miR-193a in AGS cells treated with **(F)** epigenetic modifiers (DNMT inhibitor, 5aza, and/or HDAC inhibitor, TSA), **(G)** shRNA against STAT3 (shSTAT3), or **(H)** a specific STAT3 inhibitor (JSI-124). Each bar represents mean ± SD of duplicate experiments (*p < 0.05, **p < 0.01; ***p < 0.005).

To identify genes that were epigenetically silenced by STAT3 activation, we performed a sequencing based method (MBDCap-Seq) in AGS gastric cancer cell line (an adenocarcinoma with constitutive STAT3 activation), depleted of STAT3 by viral-based shRNA (9), to compare methylation changes in this cell and the control cells (**Figure 1A**). There were 1,107 genes showing hypomethylation in AGS/shSTAT3 cells, as compared to control (AGS/shGFP), including GATA6 in which its promoter methylation has already been confirmed in AGS gastric cancer cells and patients samples in our previous study (9). Out of these 1,107 genes, 142 genes predicted to have at least one STAT3 binding elements (SBE, **Supplementary Table S1**). Interestingly, *miR-193a*, with two predicted SBE (**Figure 1B** upper panel), was found to be among our list of 1,107 genes. Although previous studies, including ours, have described the epigenetic control and function of *miR-193a* in several human cancers (20–22), its role in gastric cancer is less explored. We therefore set to examine the functional role of *miR-193a* in gastric cancer.

Bisulfite pyrosequencing was first performed to validate our sequencing results, showing a hypermethylation at *miR-193a* promoter region in AGS control (AGS/shGFP), while a decrease of methylation level at the same region was observed in AGS cells depleted of STAT3 (AGS/shSTAT3, **Figure 1B** lower panel). Next, we first investigated the role of STAT3 in the epigenetic silencing of miR-193a. Unexpectedly, ChIP-PCR (**Figure 1C**) revealed that STAT3 did not bind to either SBE1 or SBE2, as compared to control region (Ctrl, 400 bp upstream of miR-193a TSS); while STAT3 binding was noted in GATA6, as we previously described (9).

To further investigate the expression and methylation of *miR-193a*, a lower expression of *miR-193a* was observed in AGS, as well as Kato III and MKN28 gastric cancer cell lines (**Figure 1D**). In agreement with expression data, hypermethylation of miR-193a promoter was only observed in these cell lines (**Figure 1E**). Combination treatment by DNMT inhibitor (5-aza) and HDAC inhibitor (TSA) restored *miR-193a* expression (**Figure 1F**). Importantly, depletion of STAT3 (**Figure 1G**) and treatment of a STAT3 inhibitor, JSI-124 (**Figure 1H**) (23) could also restore *miR-193a* expression. Taken together, our results demonstrated that *miR-193a* was silenced by STAT3-mediated epigenetic mechanism in gastric cancer cells.

### Overexpression of miR-193a Inhibits Proliferation and Migration in AGS Cells

**Figure 2:**
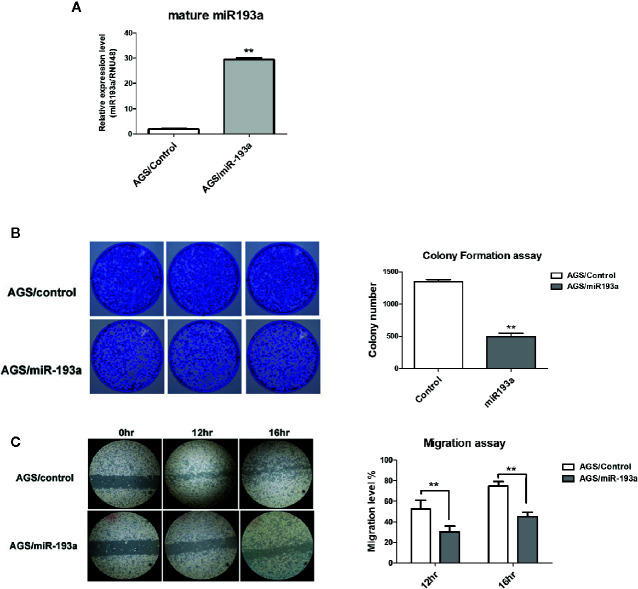
Overexpression of miR-193a inhibits cell proliferation and migration in AGS gastric cancer cells. AGS cells were transfected with control or miR-193a overexpressing plasmid. **(A)** Relative expression of mature miR-193a in control (AGS/Control) or miR-193a (AGS/miR-193a) overexpressing AGS cells, as determined by quantitative real-time PCR. Proliferation and migration of AGS/Control and AGS/miR193a cells were determined by **(B)** colony formation assay and **(C)** wound healing assay. Right panel shows the quantitative analysis of the assay. Each bar represents mean ± SD of duplicate experiments (**p < 0.01).

To examine the function of *miR-193a* in gastric cancer, *miR-193a* was overexpressed in AGS cell line (**Figure 2A**). Overexpression of *miR-193a* in AGS cells reduced proliferation in colony formation assay (**Figure 2B**) and repressed migration in wound healing assay (**Figure 2C**). These results suggested that *miR-193a* might be a tumor suppressor in gastric cancer.

### YWHAZ Is a Novel Target Gene of miR-193a in Gastric Cancer

**Figure 3:**
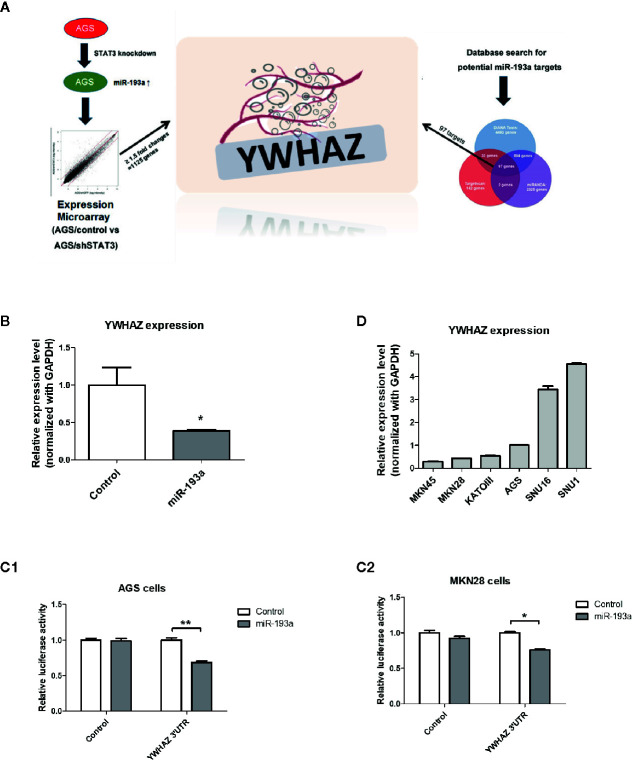
Identification of YWHAZ as a novel miR193a target gene in gastric cancer. **(A)** Schematic diagram showing the experimental scheme of this study. AGS knockdown control (AGS/control) or STAT3 depleted cells (AGS/shSTAT3) were used to perform Illumina expression microarray. The scatter plot shows the fluorescence signal of each gene on the array in AGS/control vs AGS/shSTAT3. There were 1,125 genes showing expression changes of ≥1.5-fold. In additional, bioinformatic analysis using three microRNA databases was performed to predict potential miR-193a targets. There are 97 potential miR-193a targets. YWHAZ, a potential miR-193a target showing downregulation in AGS/shSTAT3 cells (as compared to control) and has been shown to be involved in metastasis, was selected for further analysis. **(B)** Relative expression of YWHAZ in AGS gastric cancer cells overexpressed with control or miR-193a-expressing plasmid. 3’UTR luciferase confirmed that miR-193a targets YWHAZ in **(C1)** AGS and **(C2)** MKN28 cells. **(D)** Relative expression of YWHAZ in a panel of gastric cancer cell lines. Each bar represents mean ± SD of duplicate experiments (*P < 0.05, **p < 0.01).

Next, we aim to identify genes that are repressed by *miR-193a* and involved in the metastasis of gastric cancer, expression microarray was performed to identify genes showing at least 1.5-fold change of RNA expression in AGS/shSTAT3 cell line, as compared to the knockdown control. There were 1,125 genes with expression change, either upregulation or downregulation in STAT3 knockdown AGS cells (**Figure 3A**). Combination of three microRNA databases found that there are 97 common targets with predicted miR-193a binding site. One of the downregulated targets, *YWHAZ*, which has been previously found to be involved in metastasis (24, 25), was chosen for further investigation.

We then overexpressed miR-193a in AGS cells, resulting in downregulation of YWHAZ, as compared to transfection control (**Figure 3B**). Further 3’UTR luciferase assay confirmed that *miR-193a* targeted *YWHAZ* mRNA in AGS and MKN28 cells (**Figure 3C**). Taken together, these results suggested that *miR-193a* targets *YWHAZ* in gastric cancer cell lines.

### YWHAZ Enhances Invasion in Gastric Cancer Cell Line

To validate function of *YWHAZ*, we then examined the expression of *YWHAZ* in a panel of gastric cancer cell lines. Expression of *YWHAZ* partially coincided with the expression of *miR-193a* in those cancer cell lines, such that MKN45 cells showing higher expression of *miR-193a* also showed lowest expression of *YWHAZ*; while AGS cells showing modest expression of *miR-193a* and a relatively higher expression of *YWHAZ* (**Figures 1C** and **3D**). These two cell lines were then chosen for further functional validation of *YWHAZ*.

**Figure 4:**
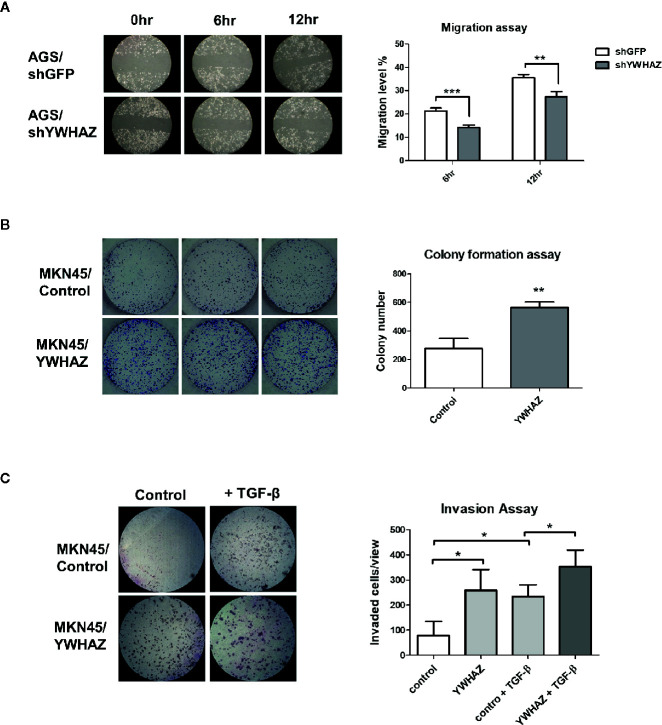
YWHAZ regulates proliferation, migration and invasion in gastric cancer cells. **(A)** Migration of AGS cells were infected with shRNA against GFP (shGFP, control) or YWHAZ (shYWHAZ) in AGS gastric cancer cells, as determined by wound healing assay. Right panel shows the quantitative analysis of the assay. MKN45 transfected control (pcDNA, MKNB45/Control) or YWHAZ expressing plasmid (MKN45/YWHAZ), were used to perform **(B)** colony formation assay, and **(C)** transwell invasion assay. Addition of TGF-β further enhanced the invasion ability of YWHAZ-overexpressing MKN45 cells. Quantitative analysis was shown in the right panel. Each bar represents mean ± SD of duplicate experiments (*P < 0.05, **p < 0.01; ***p < 0.005).

We first depleted *YWHAZ* in AGS cells, showing a reduced migration ability as compared to knockdown control (**Figure 4A**). For reciprocal experiments, overexpression of *YWHAZ* increased proliferation (**Figure 4B**) and invasion in MKN45 (**Figure 4C**). Interestingly, overexpression of *YWHAZ* further enhanced the ability of TGF-β-induced invasion in MKN45 cells. Taken together, these results suggested that *YWHAZ*, a *miR-193a* target, induced cell proliferation and invasion in gastric cancer cell lines.

### YWHAZ Increases Metastasis of Gastric Cancer Cell *In Vivo*

**Figure 5:**
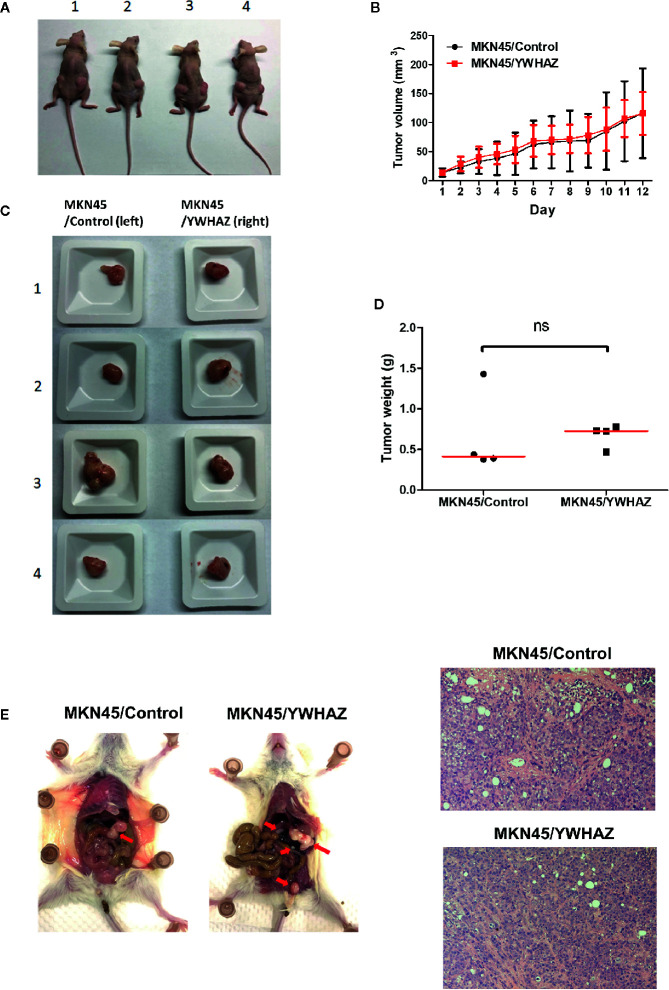
YWHAZ enhanced metastasis in MKN45 cells *in vivo*. MKN45 cells transfected with control (MKN45/Control) or plasmid expressing YWHAZ (MKN45/YWHAZ) were injected subcutaneously (s.c.) into nude mice **(A–D)** or intraperitoneally into NOD-SCID mice **(E)**. **(A)** Representative images showing the tumor in an s.c. xenograft mouse model. Overexpression of YWHAZ demonstrated a similar tumor size **(B, C)** and weight **(D)** of the xenograft, as compared to control. **(E)** However, MKN45/YWHAZ cells demonstrated more intraperitoneal nodules (red arrow) as compared to control, after 5 weeks of injection (red arrow). H&E stain presented tumor cells obtained from the liver of NOD-SCID mice.

To examine the function of *YWHAZ in vivo*, we subcutaneously injected MKN45 control and *YWHAZ* overexpressing cells into athymic nude mice. Surprisingly, overexpression of *YWHAZ* in MKN45 cells had no effect on tumor size (**Figures 5A, B**) and weight (**Figures 5C, D**), as compared to vector control. Intriguingly, overexpression of *YWHAZ* resulted in a liver metastasis of MKN45 cells, intravenously injected into NOD-SCID mice (**Figure 5E**). These results suggested that *YWHAZ* may affect metastasis of gastric cancer *in vivo*.

### Hypermethylation of *miR-193a* and Expression of *YWHAZ* in Human Gastric Cancer

**Figure 6:**
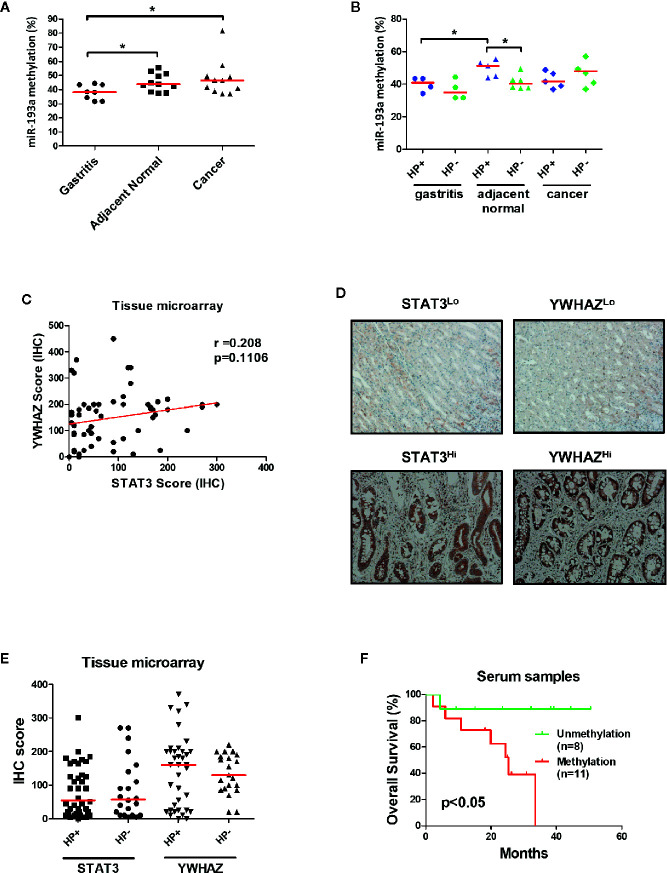
Infection of *H. pylori* is associated with miR-193a hypermethylation and increased YWHAZ expression in gastric cancer. **(A, B)** Scatter plot showing miR-193a methylation in patient tissue samples with gastritis (n = 8), and paired tumor adjacent normal and gastric cancer (n = 11). Red lines denote median. Infection of * H.pylori* is associated higher miR-193a methylation in gastritis and tumor adjacent normal. Immunohistochemistry of STAT3 and YWHAZ in gastric cancer tissue (n = 60) was performed using tissue microarray. **(C)** Scatter plot showing a positive trend between expression score of STAT3 and YWHAZ. **(D)** Tissues infected with *H. pylori* showed a higher expression of YWHAZ than those without *H. pylori* infection. **(E)** Representative photos showing low (STAT3^Lo^, YWHAZ^Lo^) and high (STAT3^Hi^, YWHAZ^Hi^) expression of STAT3 and YWHAZ in tissue microarray. **(F)** Kaplan-Meier analysis showed that patients with serum miR-193a methylation is associated with shorter overall survival as compared to samples without methylation (p < 0.05).

To confirm the role of *miR-193a* and *YWHAZ* in gastric carcinogenesis, we analyzed promoter methylation of *miR-193a* in tissue samples obtained from gastritis (n = 8), and paired tumor adjacent normal and gastric cancer (n = 11) by bisulfite pyrosequencing. Significant higher miR-193a methylation was observed in adjacent normal and cancer tissues, as compared to gastritis (**Figure 6A**). Importantly, gastritis and adjacent normal with *H. pylori* infection demonstrated a higher *miR-193a* methylation than those without *H. pylori* infection (**Figure 6B**). We also performed tissue microarray to determine the correlation between STAT3 and YWHAZ expression in gastric cancer tissue samples (n = 60), showing a positive trend of STAT3 and YWHAZ (**Figure 6C**, r = 0.208, and **Figure 6D**). Whereas, slightly higher YWHAZ expression was also found in gastric cancer tissues infected with *H. pylori* (**Figure 6E**).

Given *miR-193a* is involved in tumor metastasis, we therefore investigated if miR-193a methylation can be a biomarker for predicting patient outcomes using cell-free DNA obtained from serum samples. Gastric cancer patients with higher serum miR-193a methylation is associated with shorter overall survival than those with lower serum miR-193a methylation (**Figure 6F**, p = 0.04). Taken together, these results suggested that infection of *H. pylori* was associated with higher *miR-193a* methylation and *YWHAZ* expression, probably due to activation of JAK/STAT signaling. Methylation of *miR-193a* can be a minimal-invasive biomarker for predicting patient outcomes in gastric cancer.

## Discussion

Aberrant activation of JAK/STAT signaling could contribute to gastric carcinogenesis, partially due to alteration of the epigenome (26, 27). In the current study, by methylomic analysis, we identified that *miR-193a*, a potential STAT3 target, is epigenetically silenced by DNA methylation in gastric cancer cells. However, combination treatment of both DNMTi (5aza) and HDACi (TSA) only resulted in robust reexpression of miR-193a, suggesting that other mechanisms such as histone modifications are also responsible for the transcriptional regulation of miR-193a.

Depletion of STAT3 and treatment of STAT3 inhibitor in AGS cells partially restored miR-193a expression, suggesting that miR-193a is epigenetically suppressed by STAT3 activation. Bioinformatic analysis found that there are two STAT3 binding sites at the upstream promoter region of *miR-193a*; however, ChIP-PCR showed that STAT3 did not bind to these regions. These results suggested that epigenetic silencing of miR-193a is indirectly affected by STAT3, probably through STAT3-mediated upregulation of DNMT (28) and EZH2 (29). We have previously demonstrated that E2F6-mediated EZH2 repression is responsible for the epigenetic silencing of miR-193a in ovarian cancer. Whether E2F6 also participates in the epigenetic silencing of miR-193a requires further investigation.

Furthermore, restoration of *miR-193a* in gastric cancer cell suppressed proliferation and migration *in vitro*, probably due to suppression of YWHAZ, which is found to be a target of miR-193a. In this regard, epigenetic silencing of miR-193a could result in upregulation of YWHAZ and tumor invasion *in vitro* and *in vivo*. As *YWHAZ* was previously found to be involved in cancer metastasis (24, 25), overexpression of *YWHAZ* could enhance gastric cancer invasion *in vitro* and *in vivo*. Clinically, higher *miR-193a* methylation, and *YWHAZ* expression, was observed in patient tissue samples with *H. pylori* infection, suggesting that *H. pylori* mediated STAT3 activating might be responsible for this phenomenon. Interestingly, miR-193a methylation can also be served as a biomarker for predicting patient outcomes in serum of gastric cancer patients.

YWHAZ, also knowns as 14-3-3ζ, belongs to the 14-3-3 protein family and are highly conserved regulatory proteins in both plants and mammals (30, 31). 14-3-3 proteins participate in a wide range of signaling pathways through binding to specific phosphoserine/phosphothreonine (pSer/Thr) containing motifs in target protein (32). A critical role of 14-3-3 protein family has been described in breast, lung, and head and neck cancers, suggesting that YWHAZ plays a pro-oncogenic role in multiple tumor types (33). Previous studies also showed that YWHAZ could form a complex with β-catenin to activate Wnt pathway, thus enhancing metastatic potentials in cancers (34, 35). This result is in agreement with our study that YWHAZ could increase metastasis *in vitro* and *in vivo*, probably through STAT3-mediated epigenetic silencing of miR-193a.

Several studies showed that *miR-193a* plays an important role in the progression of human cancer, such as lung and colorectal cancer (36, 37). Studies found that miR-193a could be sponged and suppressed by long non-coding RNA, *via* a competing endogenous RNA (ceRNA) mechanism, resulting in the proliferation of gastric cancer cells (16). This phenomenon may partially explain the in-concordance between the expression level of *miR-193a* and *YWHAZ* in some of the cell lines. Specifically, high level of *miR-193a* in SNU1 and SNU16 cells may be suppressed by ceRNA mechanism resulting in a higher expression of YWHAZ in these cells. However, further experiment is required to confirm this phenomenon.

In conclusion, aberrant JAK/STAT signaling might participate in the epigenetic silencing of miR-193a, *via* promoter hypermethylation, in gastric cancer. Suppression of *miR-193a* might induce YWHAZ overexpression, resulting in gastric cancer metastasis. Inhibition of STAT3 could restore miR-193a expression and subsequent inhibition of metastasis. Methylation of miR-193a could also act as a biomarker for the prediction of patient outcome. The therapeutic potential of targeting STAT3 in the treatment of gastric cancer deserves further investigation.

## Data Availability Statement

The datasets presented in this study can be found in online repositories. The names of the repository/repositories and accession number(s) can be found below: the NCBI Gene Expression Omnibus (GSE154080).

## Ethics Statement

The studies involving human participants were reviewed and approved by the Institutional Review Board (IRB) of the Chang Gung Memorial Hospital, Chiayi, Taiwan. The patients/participants provided their written informed consent to participate in this study. The animal study was reviewed and approved by Animal Experimentation Ethics Committee of the National Chung Cheng University.

## Author Contributions

J-LC, Y-CC, J-LL, J-TL, and PY performed experiments. Y-MC and JL performed the bioinformatic analysis. K-LW, T-SC, W-MC, Y-YH, and C-SW collected patient samples. J-LC, G-LL, and MC wrote the manuscript. S-FW, M-KC, CL, and MC performed experimental design. All authors contributed to the article and approved the submitted version.

## Funding

This study was supported by grants from Chang Gung Memorial Hospital, Taiwan (CORPG6D0031~33 and CORPG6G0031~32), the Ministry of Science and Technology, Taiwan (MOST 108-2314-B-194-003-MY2), National Institutes of Health, USA (P30CA16058 to The Ohio State University Comprehensive Cancer Center), National Institutes of Health, USA (R50CA211524 to PY), and the Center for Innovative Research on Aging Society (CIRAS) from The Featured Areas Research Center Program within the framework of the Higher Education Sprout Project by Ministry of Education (MOE) in Taiwan.

## Conflict of Interest

The authors declare that the research was conducted in the absence of any commercial or financial relationships that could be construed as a potential conflict of interest.
